# Extending the game immersion questionnaire to online users

**DOI:** 10.3389/fpsyg.2025.1473821

**Published:** 2025-02-11

**Authors:** Valērijs Dombrovskis, Jeļena Ļevina, Aleksejs Ruža

**Affiliations:** ^1^Business Department, RISEBA University of Applied Sciences, Riga, Latvia; ^2^Latvian Prison Administration, Riga, Latvia; ^3^Psychology Department, Daugavpils University, Daugavpils, Latvia

**Keywords:** digital immersion, levels of immersion, engagement, engrossment, total immersion, online users

## Abstract

**Introduction:**

The Game Immersion Questionnaire (GIQ) serves as a tool for evaluating immersion within the realm of online gaming. Immersion is a crucial psychological state experienced by users interacting with digital environments, influencing their engagement, engrossment, and total involvement. While the original GIQ was structured around seven first-order factors and three second-order factors, its applicability beyond gaming contexts remains unexplored. This study extends the GIQ to capture the immersion experiences of a more expansive cohort of online users beyond gamers.

**Methods:**

The extended GIQ was administered to a cohort of active internet users from Latvia (*n* = 227), aged 13 to 65 (M = 26.6, SD = 8.4), with 53.7% identifying as female, 33% as male, and 13.2% as nonbinary. Factor analysis was conducted to examine the structure of the extended questionnaire and validate its applicability to a broader range of online users.

**Results:**

Factor analysis revealed three first-order factors – Engagement, Engrossment, and Total Immersion – that together form a global second-order factor named the Level of Immersion in the Digital Environment. These findings align with the original hierarchical structure of the GIQ while extending its relevance to a wider population of online users.

**Discussion:**

The extended GIQ provides insights into the immersion experiences of a diverse range of online users across various digital environments. By adapting the GIQ for broader use, this study contributes to the understanding of immersion beyond gaming and supports its application in different online contexts.

## 1 Introduction

The digital environment constitutes an integral component of contemporary social dynamics, where interactions among online users and the consumption of digital information as a primary source of social knowledge have rapidly become central to societal functioning. In the field of cyberpsychology, the term “immersion” has gained recognition, particularly in delineating experiences within video gaming contexts. It is noteworthy that immersion is conceptualized as a suboptimal and non-extreme state, distinct from the flow state (Csikszentmihalyi, 1990), which characterizes an individual’s intense absorption in an activity. In the context of gaming, individuals can be significantly immersed without necessarily experiencing flow during the gaming process (Cheng et al., 2015).

Extending the conceptual boundaries beyond gaming, we argue that the notion of immersion retains its relevance, providing a framework to elucidate the degrees of engagement exhibited by online users within the expansive digital environment. Consequently, immersion in the digital environment emerges as a pivotal dimension influencing the overall experiences of online users.

### 1.1 The levels of immersion in the digital environment

Immersion, as defined above, entails a state of profound mental involvement, potentially leading to a disassociation from the awareness of the physical world, driven by a shift in attentional focus (Agrawal et al., 2019), though this is not obligatory. Various levels of involvement can coexist.

The impact of immersion in the digital environment on users’ experience is profound. When immersed, users establish a deep connection with the digital environment, experiencing a sense of presence and diminished self-awareness, akin to being within the digital world itself (Huang et al., 2020). This heightened engagement yields positive outcomes, including increased motivation, enjoyment, and a sense of accomplishment.

Conversely, a lack of immersion leads to a less positive digital experience (Guerra-Tamez, 2023). Users may feel disconnected, disinterested, and exhibit reduced motivation to participate, limiting their ability to fully comprehend and engage with the digital environment. Overall, the level of immersion emerges as a pivotal factor influencing users’ experience and their connection to the digital environment (Nah et al., 2022).

**Figure 1:**
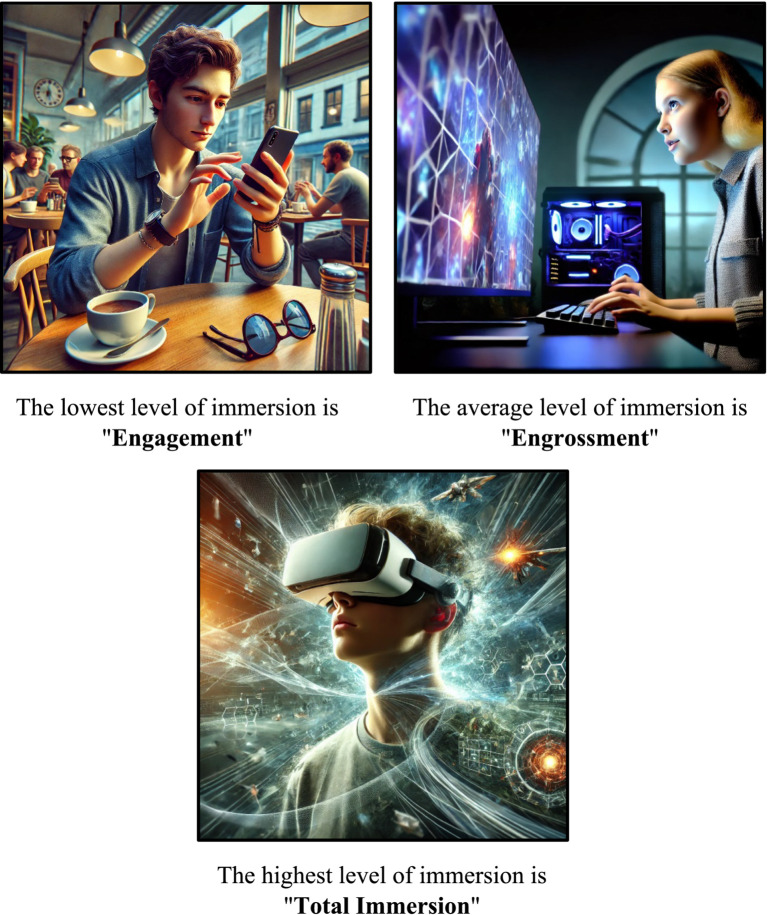
Immersion levels.

In the exploration of involvement stages in gaming (Brown and Cairns, 2004), three distinct levels have been identified: engagement, engrossment, and total immersion. These levels are scrutinized within samples of online gamers (refer to Figure 1).

- *Engagement* represents the initial and lowest level of involvement. Users experiencing engagement in the digital environment may exhibit a low level of interaction and involvement in the digital space, while still maintaining awareness of their physical surroundings.
- *Engrossment* marks the subsequent stage of involvement, characterized by a heightened emotional investment in the digital environment. Users at this level are less aware of their surroundings, with attention and emotions directly influenced by the digital environment, fostering emotional attachment to the digital environment.
- *Total immersion* signifies the pinnacle, equating to a state of presence where the digital environment takes precedence. Users in a state of total immersion are wholly absorbed in the digital environment and are detached from the physical world around them.

The psychological and emotional state of a user within the digital environment is significantly impacted by the experienced level of immersion. These factors, in turn, influence a user’s behavior and actions within the digital environment, ultimately shaping the impact of a digital identity on human development.

The categorization of immersion levels in the digital environment serves to provide a framework for comprehending the diverse stages of involvement and connection individuals may undergo while interacting within digital environments (Lombard and Ditton, 1997). This knowledge not only informs the design of digital environments but also contributes to the development of technologies that foster positive experiences and well-being while minimizing potential negative effects.

### 1.2 The connection between immersion and self-transformation

The relationship between immersion and self-transformation underscores the complex and multifaceted nature of how an individual’s level of immersion in the digital environment can influence their process of self-transformation. High levels of immersion, particularly in cases of total immersion, are often associated with significant changes in self-concept and digital identity. However, this relationship is not absolute; rather, it involves a dynamic interaction between the user’s psychological state and the attributes of the digital environment (McCreery et al., 2013).

It is important to note that immersion does not uniformly lead to self-transformation. While users experiencing total immersion may undergo profound changes in their self-concept, those with lower levels of immersion, such as engagement or engrossment, may still experience meaningful, albeit less intense, forms of self-representation adjustment. Limited immersion, characterized by low levels of involvement, may reduce the likelihood of significant self-transformation but can still contribute to minor shifts in self-perception and digital behavior (Tombul and Sari, 2021).

The stages of immersion in the digital environment delineate a progressive and iterative process of engagement. As users move from engagement to engrossment and eventually reach total immersion, their self-concept may gradually integrate with their digital identity. This process can result in varying degrees of self-transformation, depending on factors such as individual personality traits, the immersive potential of the digital environment, and the nature of the digital activities involved (Kim et al., 2014).

Research highlights the importance of individual differences in shaping the relationship between immersion and self-transformation. For instance, users with a predisposition toward imaginative involvement or those who seek intense digital experiences are more likely to experience deeper levels of immersion and corresponding self-transformation (Bailenson et al., 2008). Conversely, users who engage in digital environments primarily for informational or functional purposes may exhibit lower immersion levels, resulting in a more stable self-concept (Zimmermann et al., 2023).

The connection between immersion and self-transformation should be viewed as a correlation influenced by various moderating factors, rather than a straightforward cause-and-effect relationship. This nuanced perspective allows for a better understanding of how digital environments contribute to the evolving nature of users’ self-concepts and digital identities.

### 1.3 The rationale for extending the GIQ beyond gaming

Given the factors mentioned above, extending the GIQ to serve as a tool for assessing immersion among a broader range of online users has become a crucial endeavor for advancing research in this field. Originally, the GIQ was developed to evaluate immersion in gaming environments, where the immersive experience is closely tied to psychological engagement and motivations for gameplay. Recent research on immersion in video games highlights the complex interplay of psychological factors driving player involvement, which further underscores the relevance of GIQ for capturing different dimensions of user engagement (Gursesli et al., 2024).

While the GIQ, designed by Cheng et al. (2015), has significantly contributed to understanding immersion in gaming, its applicability to a broader spectrum of online users requires critical examination. The GIQ, structured around seven first-order factors and three second-order factors, was instrumental in delineating dimensions like Engagement, Engrossment, and Total Immersion, specifically tailored for the gaming community. However, the immersion experiences of online users extend beyond gaming, encompassing diverse digital activities, such as social interactions, informational browsing, and educational engagements, where users may exhibit distinct immersion patterns.

Extensive research on immersion in digital environments has underscored the importance of developing reliable instruments for its measurement. The GIQ has been widely recognized for its robustness and internal consistency in capturing different levels of immersion (Cheng et al., 2014). Beyond gaming, other tools have been introduced to measure immersion in various contexts, such as educational games and augmented reality applications (Georgiou and Kyza, 2017; Nagalingam et al., 2020). These instruments account for the unique characteristics of user interaction with digital environments, including visual design, the balance between challenge and skill, and emotional engagement.

Instruments such as the ARI Questionnaire and EDUGXQ were specifically designed for augmented reality applications and educational games, respectively, providing valuable insights into immersion in these specialized contexts (Georgiou and Kyza, 2017; Nagalingam et al., 2020). The miniPXI questionnaire offers a concise method for evaluating player experience in gaming environments (Haider et al., 2022). While these tools have demonstrated effectiveness in their respective domains, their applicability to a broader range of online experiences remains limited due to their context-specific design.

In contrast, the GIQ provides a more versatile framework for measuring immersion across various digital platforms. Its hierarchical structure, encompassing first-order factors such as Engagement, Engrossment, and Total Immersion, allows for a nuanced understanding of user experiences in diverse digital environments. Unlike instruments tailored for specific applications, the GIQ’s design enables its adaptation to different digital contexts, making it particularly suitable for studying the immersion of online users beyond gaming.

Studies have further confirmed that immersion follows a hierarchical structure consisting of multiple levels of involvement, each characterized by varying degrees of absorption and emotional attachment (Brown and Cairns, 2004; Ermi and Mäyrä, 2005). The validation of such instruments typically involves exploratory and confirmatory factor analyses to establish their construct validity (Cheng et al., 2014).

This study focuses on extending the GIQ by testing it on a wider sample of online users. By hypothesizing a hierarchical structure of immersion – including first-order factors such as Engagement, Engrossment, and Total Immersion – and a global second-order factor, the Level of Immersion in the Digital Environment, the research addresses the nuanced dimensions of immersion among online users. This approach aligns with the overarching model proposed by Cheng et al. (2015), emphasizing the interplay and correlations among these fundamental factors. Through this extension, a more comprehensive understanding of immersion in the digital environment for a diverse range of online users is provided.

To empirically validate the extended GIQ and better understand the immersive experiences of online users, several hypotheses were formulated based on prior theoretical models and research findings. These hypotheses address the key dimensions of immersion, including engagement, engrossment, and total immersion, as well as their hierarchical structure. The following section outlines these hypotheses and provides the rationale for each.

### 1.4 The hypothesis development

This study aims to investigate the immersive experiences of online users by extending the Game Immersion Questionnaire (GIQ) to a broader range of digital environments. Based on previous research and theoretical models, the following hypotheses were formulated:

H1: Higher levels of engagement are positively associated with engrossment in digital environments.

Rationale: engagement represents the initial stage of immersion, where users become interested and involved in a digital experience. Previous studies have indicated that increased engagement often leads to engrossment as users develop a deeper connection with the digital environment (Brown and Cairns, 2004; Michailidis et al., 2018).

H2: Higher levels of engrossment are positively associated with total immersion.

Rationale: Engrossment is characterized by emotional attachment and decreased perceptions of the external environment. Theoretical models suggest that engrossment is a prerequisite for achieving total immersion, where users feel fully absorbed in the digital space (Cheng et al., 2014).

H3: The adapted GIQ maintains a hierarchical structure, with engagement, engrossment, and total immersion forming a global second-order factor representing the level of immersion.

Rationale: The original GIQ demonstrated a hierarchical structure with three second-order factors. This hypothesis posits that the adapted version will preserve this structure when applied to online users interacting across diverse digital platforms (Cheng et al., 2014).

## 2 Method

### 2.1 Participants

The sample comprised 227 inhabitants of Latvia aged from 13 to 65 years old (M = 26.6, SD = 8.4). With regard to the statement “My Cyber Me was created in…,” the responses were categorized as follows: (1) Online games (33.9%), (2) Social media platforms (e.g., Instagram, Facebook, Twitter) (48.5%) and (3) Video hosting sites (e.g., YouTube, TikTok) (17.6%).

### 2.2 Materials and measures

The GIQ, originally designed to assess immersive experiences in video games, has been expanded to capture immersion among a broader spectrum of online users. This tool, now structured around the three core dimensions of Engagement, Engrossment, and Total Immersion, was further refined into specific factors including attraction, time investment, usability, emotional attachment, decreased perceptions, presence, and empathy.

The original GIQ was developed through a meticulous two-stage process that included item generation, scale construction, and questionnaire validation. The instrument initially comprised seven first-order factors: Emotional Involvement, Cognitive Involvement, Realism, Sensory Involvement, Control, Challenge, and Social Presence. These factors were grouped into three second-order factors: Engagement, Engrossment, and Total Immersion. Validation of the GIQ involved exploratory and confirmatory factor analyses, which confirmed the hierarchical structure of immersion. The reliability of the original scale was demonstrated through high internal consistency, with Cronbach’s *α* values exceeding 0.80 for most factors (Cheng et al., 2014). Factor loadings for the original version ranged from 0.67 to 0.89, indicating strong associations between the items and their respective factors.

In the current study, the GIQ was adapted to accommodate a broader population of online users who maintain digital identities across various digital environments. The term “avatar” was substituted with “Cyber Me,” and the scale items were revised to reflect a broader digital landscape. This terminology shift aimed to introduce a more versatile and inclusive concept that aligns better with the expanded audience. Unlike “avatar,” which often implied a specific representation, “Cyber Me” accommodates a wider range of digital identity representation strategies. This terminology allows for the representation of digital identities that have evolved beyond those traditionally used in gaming (Dombrovskis and Berga, 2021).

Additionally, the term “Game” was replaced with “digital space” to reflect the expanded scope of the study, which now includes online users who assess their level of immersion in social media platforms and video hosting sites. This modification was necessary to ensure that the instrument accurately captured the immersive experiences of users across various online platforms, beyond the context of gaming.

Table 1 shows the version of the Game Immersion Questionnaire (GIQ) that was used to assess online users’ immersion, highlighting the adapted factors and revised terminology.

**Table 1:** Items for game immersion questionnaire.

Item	Game immersion questionnaire (Cheng et al., 2015)
Engagement	
A1	I would like to spend more time in the digital space
A2	I like the appearance and style of the digital space
A3	I like being in the digital space because it is new and interesting
A4	Generally, I can handle all the difficulties associated with being in the digital space
A5	It is easy for me to control all the processes of the digital space
A6	The user interface of the digital space makes me feel comfortable
A7	I like the type of the digital space
A8	I would like to spend time collecting the information of the digital space and discussing it with friends
A9	The time I spend being in the digital space is always more than I expected
Engrossment	
B1	My ability to perceive the environment surrounding me is decreased while I am in the digital space
B2	I am impatient when someone interrupts me when I am in the digital space
B3	When I am in the digital space, I often cannot hear people who call me
B4	I often feel nervous or excited because of the digital space
B5	I often forget the passage of time while I am in the digital space
B6	It frequently happens that I forget my schedule and/or to-do things in the real world while I am in the digital space
B7	While I am in the digital space, I feel unhappy if someone interrupts me
Total immersion	
C1	While I am in the digital space, it seems to me that everything that happens there, happens to me
C2	My consciousness completely transfers from the real world to the digital space while I am solving the problems or tasks in the digital space
C3	I lose the perception of time and the real world surrounding me as if everything just stops
C4	I feel happy or sad depending on what happens to my Cyber Me and sometimes I even feel that it exists
C5	I used to be so integrated into the Cyber Me in the digital space that I could feel his/her feelings
C6	All of my senses, including vision, learning, and my mind, are concentrated on and engaged in the digital space
C7	I lose the ability of perceiving the surroundings around me; however, it seems natural for me to be totally immersed in the atmosphere of the digital space
C8	I used to feel that the Cyber Me in the digital space is controlled by my will, and not by the mouse or the keyboard, so that the Cyber Me does just what I want to do. It seems like the thoughts and consciousness of the Cyber Me and Me are connected

Responses are made on a 5-point Likert scale (from 1 – strongly agree to 5 – strongly disagree).

### 2.3 Procedure

To achieve a thorough and culturally attuned research methodology, the Game Immersion Questionnaire (GIQ) originally authored by Cheng et al. (2015), was carefully translated into Latvian. The translation was undertaken by linguists fluent in both English and Latvian, and was subsequently reviewed by a third expert who ensured the translation’s accuracy and appropriateness for the cultural context.

#### 2.3.1 Inclusion and exclusion criteria

Participants were included in the study if they met the following criteria:

- They were active users of digital technologies, such as social media platforms or online games.
- They possessed a digital identity (*Cyber Me*), which they actively used for communication and interaction with other users. Examples of “*Cyber Me*” include digital profiles, video game characters, and online avatars.
- Their “Cyber Me” had to include a visual component (e.g., an image, picture, avatar, or any other visual representation) that they perceived during interactions with other users.

Participants were excluded if:

- They did not possess a “*Cyber Me*” with a visual component.
- They were not active users of digital technologies or did not meet the specified criteria for digital interaction.
- They were unable to provide informed consent or did not complete the questionnaire.

After the translation process was completed, the recruitment of participants commenced, adhering to the inclusion and exclusion criteria. All participants gave informed and voluntary consent.

The demographic of participants was varied, including online gamers engaged in well-known games such as World of Warcraft, Albion Online, and Rust, as well as notable bloggers and influencers from Latvia. Participants were recruited using announcements on the Discord platform for gamers and through social media and video hosting platforms for bloggers. Details about prominent individuals were derived from the “AoR Indekss 2022,” with personalized outreach undertaken.

Before participating, individuals were briefed on the concept of “Cyber Me”—a digital representation in online settings, which could be a digital profile, online game character, avatar, or any similar digital entity used for interaction. They were advised that their “Cyber Me” should have a visual aspect, such as an image or icon, recognized during digital interactions (Dombrovskis et al., 2024).

Participants with multiple digital identities were instructed to select the one that most closely represented their authentic self, regardless of how it compared to their physical-world identity.

The questionnaire was hosted on Google Forms and distributed to participants, who filled it out online. Data collection spanned from October 12, 2022, to January 16, 2023. The sample size of 227 participants was determined to be sufficient based on established guidelines for conducting exploratory and confirmatory factor analysis. According to research, a minimum sample size of 150 is generally recommended for factor analysis when communalities are high and factors are well-defined (MacCallum et al., 2001). Moreover, a sample size exceeding 200 is often considered adequate for achieving stable factor solutions and reliable psychometric validation (Black and Babin, 2019). Given that the adapted version of the GIQ involved multiple first-order and second-order factors, a sample size of 227 participants provides sufficient power for detecting meaningful relationships among the variables and ensuring the robustness of the results. Detailed instructions and additional relevant information were included in the questionnaire, located in Appendix 1 of the article. Participants were urged to thoroughly review this material before completing the questionnaire.

## 3 Results

First, the principal component method was utilized separately for the three scales corresponding to Engagement, Engrossment, and Total Immersion. These scales were tested on a broader sample of online users.

### 3.1 Engagement

KMO = 0.754, χ^2^ (36) = 399.55, *p* = 0.000.

In this study, we obtained a factor structure that differs from that in the original study, as the scale was tested on a broader sample of online users, not just online gamers. The results are presented in Table 2.

**Table 2:** The results of principal component analysis for the scale “engagement”.

	Factor loadings	
Item	*C1*	*C2*
Engagement 7: I like the type of the digital space	**0.70**	−0.15
Engagement 1: I would like to spend more time in the digital space	**0.67**	0.21
Engagement 2: I like the appearance and style of the digital space	**0.66**	0.29
Engagement 8: I would like to spend time collecting information of the digital space and discussing it with friends	**0.64**	0.26
Engagement 6: The user interface of the digital space makes me feel comfortable	**0.63**	−0.19
Engagement 5: It is easy for me to control all the processes of the digital space	**0.60**	−0.56
Engagement 4: Generally, I can handle all the difficulties associated with being in the digital space	**0.51**	−0.27
Engagement 3: I like being in the digital space because it is new and interesting	**0.48**	0.45
Engagement 9: The time I spend being in the digital space is always more than I expected	−0.02	**0.69**
Eigenvalues	3.03	1.29

The bold values represent factor loadings that meet the threshold for significance in the principal component analysis, indicating strong associations between items and their respective factors.

As the second component includes the only item (which cannot form the whole scale)—Engagement 9: The time I spend being in the digital space is always more than I expected – it was deleted from the following principal component analysis.

The results of the principal component analysis without item 9 are presented below. KMO = 0.756, χ^2^ (28) = 388.38, *p* = 0.000 (see Table 3).

**Table 3:** The results of principal component analysis for the scale “engagement” without item 9.

	Factor loadings	
Item	*C1*	*C2*
Engagement 7: I like the type of the digital space	**0.70**	−0.25
Engagement 1: I would like to spend more time in the digital space	**0.67**	0.33
Engagement 2: I like the appearance and style of the digital space	**0.66**	0.35
Engagement 8: I would like to spend time collecting information of the digital space and discussing it with friends	**0.64**	0.22
Engagement 6: The user interface of the digital space makes me feel comfortable	**0.63**	−0.30
Engagement 5: It is easy for me to control all the processes of the digital space	**0.60**	−0.59
Engagement 4: Generally, I can handle all the difficulties associated with being in the digital space	**0.51**	−0.26
Engagement 3: I like being in the digital space because it is new and interesting	0.48	**0.55**
Eigenvalues	3.03	1.15

The bold values represent factor loadings that meet the threshold for significance in the principal component analysis, indicating strong associations between items and their respective factors.

As the second component now also includes only one item, Engagement 3: ‘I like being in the digital space because it is new and interesting’, which cannot form the entire scale, it was also excluded from the subsequent factor analysis. The results of the third principal component analysis without items 9 and 3 are presented below: KMO = 0.736, χ^2^ (21) = 346.72, *p* = 0.000 (see Table 4).

**Table 4:** The results of principal component analysis for the scale “engagement” without items 9 and 3.

	Factor loadings	
Item	*C1*	*C2*
Engagement 7: I like the type of the digital space	**0.72**	0.17
Engagement 1: I would like to spend more time in the digital space	**0.65**	−0.43
Engagement 6: The user interface of the digital space makes me feel comfortable	**0.65**	0.16
Engagement 2: I like the appearance and style of the digital space	**0.65**	**−0.52**
Engagement 5: It is easy for me to control all the processes of the digital space	**0.65**	**0.52**
Engagement 8: I would like to spend time collecting the information of the digital space and discussing it with friends	**0.63**	−0.25
Engagement 4: Generally, I can handle all the difficulties associated with being in the digital space	**0.52**	0.42
Eigenvalues	2.86	1.03

The bold values represent factor loadings that meet the threshold for significance in the principal component analysis, indicating strong associations between items and their respective factors.

Now two items (Engagement 2: ‘I like the appearance and style of the digital space’ and Engagement 5: ‘It is easy for me to control all the processes of the digital space’) exhibit very similar loadings on two factors. Therefore, at the next stage, factor analysis was conducted without these two items. The results of the fourth analysis without items 9, 3, 2 and 5 are presented below: KMO = 0.713, χ^2^ (10) = 165.99, *p* = 0.000 (see Table 5).

**Table 5:** The results of principal component analysis for the scale “engagement” without items 9, 3, 2 and 5.

	Factor loadings
Item	*C1*
Engagement 7: I like the type of the digital space	**0.75**
Engagement 8: I would like to spend time collecting the information of the digital space and discussing it with friends	**0.71**
Engagement 6: The user interface of the digital space makes me feel comfortable	**0.66**
Engagement 1: I would like to spend more time in the digital space	**0.63**
Engagement 4: Generally, I can handle all the difficulties associated with being in the digital space	**0.53**
Eigenvalues	2.19

The bold values represent factor loadings that meet the threshold for significance in the principal component analysis, indicating strong associations between items and their respective factors.

A single component with high factor loadings was obtained, which can be interpreted as Engagement.

Additionally, internal consistency of the scale was checked. Cronbah’s ɑ was 0.67.^1^

During the factor analysis of the Engagement factor, several items were eliminated due to their low or inconsistent factor loadings or limited applicability in the context of online users. Specifically, the following items were excluded: A2 “I like the appearance and style of the digital space,” A3 “I like being in the digital space because it is new and interesting,” A5 “It is easy for me to control all the processes of the digital space,” A7 “I like the type of the digital space,” and A9 “The time I spend being in the digital space is always more than I expected.” These items, which were originally designed to capture user engagement in gaming environments, were deemed less relevant for online users interacting across diverse digital platforms, where engagement may be driven by different factors.

### 3.2 Engrossment

Principal component analysis was conducted using the principal component method, and the results yielded a KMO of 0.783, χ^2^ (21) = 389.01, *p* = 0.000. Similar to the first scale in this study, we obtained a factor structure that differs from the original study, as this scale was tested on a broader sample of online users, not just online gamers (see Table 6).

**Table 6:** The results of principal component analysis for the scale “engrossment”.

	Factor loadings	
Item	*C1*	*C2*
Engrossment 6: It frequently happens that I forget my schedule and/or to-do things in the real world while I am in the digital space	**0.71**	−0.53
Engrossment 5: I often forget the passage of time while I am in the digital space	**0.70**	−0.44
Engrossment 3: When I am in the digital space, I often cannot hear people	**0.69**	0.09
Engrossment 2: I am impatient when someone interrupts me when I am in the digital space	**0.67**	0.50
Engrossment 1: My ability to perceive the environment surrounding me is decreased while I am in the digital space	**0.64**	−0.23
Engrossment 7: While I am in the digital space, I feel unhappy if someone interrupts me	**0.61**	**0.50**
Engrossment 4: I often feel nervous or excited because of the digital space	**0.60**	0.22
Eigenvalues	3.04	1.08

The bold values represent factor loadings that meet the threshold for significance in the principal component analysis, indicating strong associations between items and their respective factors.

Due to the item (Engrossment 7: While I am in the digital space, I feel unhappy if someone interrupts me) displaying very similar loadings on two factors, the subsequent factor analysis was conducted without this item. The results of the second principal component analysis without item 7 are presented below: KMO = 0.759, χ^2^ (15) = 316.11, *p* = 0.000 (see Table 7).

**Table 7:** The results of principal component analysis for the scale “engrossment” without item 7.

	Factor loadings
Item	*C1*
Engrossment 6: It frequently happens that I forget my schedule and/or to-do things in the real world while I am in the digital space	**0.76**
Engrossment 5: I often forget the passage of time while I am in the digital space	**0.73**
Engrossment 3: When I am in the digital space, I often cannot hear people	**0.69**
Engrossment 1: My ability to perceive the environment surrounding me is decreased while I am in the digital space	**0.66**
Engrossment 2: I am impatient when someone interrupts me when I am in the digital space	**0.63**
Engrossment 4: I often feel nervous or excited because of the digital space	**0.60**
Eigenvalues	2.76

The bold values represent factor loadings that meet the threshold for significance in the principal component analysis, indicating strong associations between items and their respective factors.

A single component with high factor loadings was identified, which can be interpreted as Engrossment.

Additionally, internal consistency of the scale was checked. Cronbah’s ɑ was 0.76.

In the Engrossment factor, item B7 “While I am in the digital space, I feel unhappy if someone interrupts me” was removed due to low factor loadings and limited relevance in the broader context of online user experiences. This item was more suitable for gaming contexts, where engrossment often involves uninterrupted concentration, and thus did not generalize well to other types of digital spaces. Excluding this item helped improve the content validity of the scale for a wider audience.

### 3.3 Total immersion

Principal component analysis was conducted using the principal component method. KMO = 0.904, χ^2^ (28) = 1050.41, *p* = 0.000. In this study, we obtained a factor structure that differs from the original study, as this scale was tested on a broader sample of online users, not just online gamers (see Table 8).

**Table 8:** The results of principal component analysis for the scale “total immersion”.

	Factor loadings
Item	*C1*
Total immersion 2: My consciousness completely transfers from the real world to the digital space while I am solving the problems or tasks in the digital space	**0.88**
Total immersion 4: I feel happy or sad depending on what happens to my Cyber Me and sometimes I even feel that it exists	**0.84**
Total immersion 1: While I am in the digital space, it seems to me that everything that happens there, happens to me	**0.82**
Total immersion 5: I used to be so integrated into the Cyber Me in the digital space that I could feel his/her feelings	**0.80**
Total immersion 7: I lose the ability of perceiving the surroundings around me; however, it seems natural for me to be totally immersed in the atmosphere of the digital space	**0.78**
Total immersion 8: I used to feel that the Cyber Me in the digital space is controlled by my will, and not by the mouse or the keyboard, so that the Cyber Me does just what I want to do. It seems like the thoughts and consciousness of the Cyber Me and Me are connected	**0.76**
Total immersion 3: I lose the perception of time and the real world surrounding me as if everything just stops	**0.68**
Total immersion 6: All of my senses, including vision, learning and my mind, are concentrated on and engaged in the digital space	**0.68**
Eigenvalues	4.90

The bold values represent factor loadings that meet the threshold for significance in the principal component analysis, indicating strong associations between items and their respective factors.

A single component with high factor loadings was derived, interpreted as “Total Immersion.” Additionally, the internal consistency of the scale was checked, resulting in Cronbach’s *α* of 0.91.

As shown in Table 9, there is a statistically significant positive correlation between Engagement and Engrossment (r = 0.13, *p* < 0.05). Additionally, there is a statistically significant positive correlation between Engagement and Total Immersion (r = 0.41, *p* < 0.001). Finally, there is a statistically significant positive correlation between Engrossment and Total Immersion (r = 0.46, *p* < 0.001).

**Table 9:** Correlations among the obtained first-order factors.

Scale	Engagement	Engrossment	Total immersion
Engagement	-	0.13*	0.41**
Engrossment		-	0.46**
Total immersion			-

* *p* < 0.05, ** *p* < 0.001.

The bold values represent factor loadings that meet the threshold for significance in the principal component analysis, indicating strong associations between items and their respective factors.

A global model comprising three obtained first-order factors (Engagement, Engrossment and Total Immersion) was analysed. Factor analysis for the obtained first-order factors was conducted using the principal component method. KMO = 0.517, χ^2^ (3) = 97.490, *p* = 0.000 (see Table 10).

**Table 10:** Results of the factor analysis.

	Factor loadings
Second order factors	*C1*
F3 total immersion	**0.87**
F2 engrossment	**0.69**
F1 engagement	**0.67**
Eigenvalues	1.69

The bold values represent factor loadings that meet the threshold for significance in the principal component analysis, indicating strong associations between items and their respective factors.

The obtained three first-order factors – Engagement, Engrossment, and Total Immersion – were originally treated as second-order factors in the Game Immersion Questionnaire (GIQ). However, in the current study, based on the results of the factor analysis, these factors demonstrated sufficiently high and distinct loadings, warranting their reinterpretation as first-order factors. This change reflects the observed structure of correlations among these factors, where each contributes directly to the global construct of immersion. Consequently, the Level of Immersion in the Digital Environment was defined as a second-order factor that encompasses these three core dimensions. Such a reconfiguration better aligns with the immersion experiences reported by online users across various digital platforms.

## 4 Discussion

This research thoroughly investigated the reliability and factorial organization of the GIQ when applied to an expanded group of online users. Aligning with foundational theories of immersion, the study corroborated the positive relationships among the various levels of immersion. Specifically, the hypotheses H1, H2, and H3 were supported by the data, confirming the hierarchical structure of immersion in digital environments (Cheng et al., 2015).

The findings indicated a positive correlation between engagement and engrossment (H1), as well as between engrossment and total immersion (H2). Furthermore, the adapted GIQ maintained a three-factor hierarchical structure (H3), consistent with prior studies on immersion in gaming contexts (Brown and Cairns, 2004; Michailidis et al., 2018). These outcomes are in accord with prior studies, which also reported positive correlations among the dimensions of the original GIQ. Together, these findings emphasize that immersion in digital contexts fundamentally comprises three primary dimensions: Engagement, Engrossment, and Total Immersion.

The structural analysis of the extended GIQ revealed a clearly delineated three-factor structure, validating the original dimensions within a more extensive context of online users. The GIQ was evaluated across a broader spectrum of participants, including those from diverse digital platforms such as social media sites and video hosting services. This wider application did not require modifications to the original instrument but rather confirmed its applicability to an expanded user base.

The identified factors aligned perfectly with the pre-established theoretical framework, confirming that digital immersion inherently involves Engagement, Engrossment, and Total Immersion. This concurs with earlier research, which validated the factorial structure of the GIQ for a broader audience (Daşdemir, 2023). The psychometric qualities of the extended GIQ, as utilized in this study, verified its reliability and functional value. The research established solid benchmarks of internal consistency and factorial validity across the GIQ’s scales.

The findings not only substantiated the expected robust internal consistency within the GIQ scales but also verified their alignment with the three distinct levels of immersion: Engagement, Engrossment, and Total Immersion. Moreover, the results established that these levels of immersion represent separate yet interrelated dimensions. While this study was based on a sample of online users, it represents a critical advancement in assessing the suitability of the extended GIQ as a tool for measuring immersion across varied digital settings.

### 4.1 Limitations

Despite the positive outcomes of the study, several limitations should be acknowledged. First, the sample consisted solely of participants from Latvia, which may limit the generalizability of the findings to other cultural contexts. Future research should include more diverse samples to enhance the external validity of the results.

Second, the reliance on self-reported data introduces the possibility of response biases, such as social desirability bias. Although efforts were made to ensure the anonymity and voluntary nature of participation, this limitation remains inherent in self-reported measures (Teh et al., 2023).

Third, while the study demonstrated the applicability of the extended GIQ across different digital platforms, it primarily focused on users with visually identifiable digital identities (“Cyber Me”). This specific criterion may exclude certain types of online users who engage with digital environments in different ways.

Lastly, the cross-sectional design of the study limits the ability to draw causal conclusions about the relationships between different levels of immersion. Longitudinal studies would be valuable in further exploring the dynamics of digital immersion over time.

## 5 Conclusion

The extended GIQ has been demonstrated to be a robust and valid instrument for measuring immersion across a wider spectrum of online users who maintain digital identities within varied digital contexts.

The findings from this study provide additional support to the theoretical framework of immersion and affirm the three-level model established by Cheng et al. (2015). These insights contribute to a deeper comprehension of the complex and multi-dimensional nature of immersion in digital environments.

Future stages in the development of the extended GIQ might encompass confirmatory factor analysis using a broader, international cohort, establishing both concurrent and convergent validity, and conducting tests for retest reliability. Further studies are recommended to explore the validation of the extended GIQ, not solely through self-report measures but also by incorporating behavioral metrics. Such methodologies could reveal underlying relationships, such as the impact of cognitive load on engagement, physiological indicators of arousal on engrossment, and the integration of personal information in the process of achieving total immersion.

The extended GIQ not only serves as an effective tool but also lays the groundwork for further investigations, providing a rich basis for delving into the complexities of immersion within the dynamically changing digital landscape.

## Data Availability

The original contributions presented in the study are included in the article/Supplementary material, further inquiries can be directed to the corresponding author.

## Footnotes

^1^As with other reliability coefficients, alpha should be above 0.70, however, it is common to see journal articles where one or more scales have somewhat lower alphas (e.g., in the 0.60–0.69 range), especially if there is only a handful of items in the scale (Leech et al., 2008).

## References

[ref1] Agrawal S. Simon A. Bech S. Bærentsen K. Forchhammer S. (2019). Defining immersion: literature review and implications for research on immersive audiovisual experiences. J. Audio Eng. Soc. 68, 404–417. doi: 10.17743/jaes.2020.0039

[ref2] Bailenson J. N. Blascovich J. Guadagno R. E. (2008). Self-representations in immersive virtual environments. J. Appl. Soc. Psychol. 38, 2673–2690. doi: 10.1111/j.1559-1816.2008.00409.x

[ref3] Black W. Babin B. J. (2019). “Multivariate data analysis: its approach, evolution, and impact” in The great facilitator: Reflections on the contributions of Joseph F. Hair, Jr. to marketing and business research (Cham: Springer International Publishing), 121–130.

[ref4] Brown E. Cairns P. (2004). “A grounded investigation of game immersion” in Proceedings of the CHI ‘04 extended abstracts on human factors in computing systems (Vienna, Austria: ACM), 1297–1300.

[ref5] Cheng M. T. She H. C. Annetta L. A. (2015). Game immersion experience: its hierarchical structure and impact on game-based science learning. J. Comput. Assist. Learn. 31, 232–253. doi: 10.1111/jcal.12066

[ref6] Csikszentmihalyi M. (1990). Flow: The psychology of optimal experience. Harper-Perennial: New York, NY.

[ref7] Daşdemir Y. (2023). Classification of emotional and immersive outcomes in the context of virtual reality scene interactions. Diagnostics 13:3437. doi: 10.3390/diagnostics13223437, PMID: 37998573 PMC10670519

[ref8] Dombrovskis V. Berga L. (2021). KIBER ES. Viss par kiberpasauli: Izdevniecība “Ezerrozes grāmatas”. Available at: https://kiberprats.lv/#pdf-kiber-es/1/

[ref9] Dombrovskis V. Ļevina J. Ruža A. (2024). Reliability and factorial validity of the cross-media self-presence questionnaire (CM-SPQ). Comput. Hum. Behav. Rep. 13:100370. doi: 10.1016/j.chbr.2024.100370

[ref10] Ermi L. Mäyrä F. (2005). Fundamental components of the gameplay experience: Analysing immersion. In Proceedings of DiGRA 2005 conference: Changing views: worlds in play.

[ref11] Georgiou Y. Kyza E. A. (2017). The development and validation of the ARI questionnaire: an instrument for measuring immersion in location-based augmented reality settings. Int. J. Hum. Comput. Stud. 98, 24–37. doi: 10.1016/j.ijhcs.2016.09.014

[ref12] Guerra-Tamez C. R. (2023). The impact of immersion through virtual reality in the learning experiences of art and design students: the mediating effect of the flow experience. Educ. Sci. 13:185. doi: 10.3390/educsci13020185

[ref13] Gursesli M. C. Martucci A. Mattiassi A. D. Duradoni M. Guazzini A. (2024). Development and validation of the psychological motivations for playing video games scale (PMPVGs). Simul. Gaming 55, 856–885. doi: 10.1177/10468781241260861

[ref14] Haider A. Harteveld C. Johnson D. Birk M. V. Mandryk R. L. Seif El-Nasr M.. (2022). miniPXI: Development and validation of an eleven-item measure of the player experience inventory. Proceedings of the ACM on human-computer interaction, 6(CHI PLAY), 1–26.

[ref15] Huang C. L. Luo Y. F. Yang S. C. Lu C. M. Chen A. S. (2020). Influence of students’ learning style, sense of presence, and cognitive load on learning outcomes in an immersive virtual reality learning environment. J. Educ. Comput. Res. 58, 596–615. doi: 10.1177/0735633119867422

[ref16] Kim K. Rosenthal M. Z. Zielinski D. J. Brady R. (2014). Effects of virtual environment platforms on emotional responses. Comput. Methods Prog. Biomed. 113, 882–893. doi: 10.1016/j.cmpb.2013.12.024, PMID: 24440136

[ref17] Leech N. L. Barret K. C. Morgan G. A. (2008). SPSS for intermediate statistics: Use and interpretation. 3rd Edn. Lawrence Erlbaum Associates: New York, NY; London, UK.

[ref18] Lombard M. Ditton T. (1997). At the heart of it all: the concept of presence. Journal of computer-mediated. Communication 3:321. doi: 10.1111/j.1083-6101.1997.tb00072.x, PMID: 39872724

[ref19] MacCallum R. C. Widaman K. F. Preacher K. J. Hong S. (2001). Sample size in factor analysis: the role of model error. Multivar. Behav. Res. 36, 611–637. doi: 10.1207/S15327906MBR3604_06, PMID: 26822184

[ref20] McCreery M. P. Schrader P. G. Krach S. K. Boone R. (2013). A sense of self: the role of presence in virtual environments. Comput. Hum. Behav. 29, 1635–1640. doi: 10.1016/j.chb.2013.02.002

[ref21] Michailidis L. Balaguer-Ballester E. He X. (2018). Flow and immersion in video games: the aftermath of a conceptual challenge. Front. Psychol. 9:1682. doi: 10.3389/fpsyg.2018.01682, PMID: 30233477 PMC6134042

[ref22] Nagalingam V. Ibrahim R. Yusoff R. C. M. (2020). EDUGXQ: user experience instrument for educational games’ evaluation. Int. J. Adv. Comput. Sci. Appl. 11, 562–569. doi: 10.14569/IJACSA.2020.0110170, PMID: 38915103

[ref23] Nah K. Oh S. Han B. Kim H. Lee A. (2022). A study on the user experience to improve immersion as a digital human in lifestyle content. Appl. Sci. 12:12467. doi: 10.3390/app122312467

[ref24] Teh W. L. Abdin E. Pv A. Siva Kumar F. D. Roystonn K. Wang P.. (2023). Measuring social desirability bias in a multi-ethnic cohort sample: its relationship with self-reported physical activity, dietary habits, and factor structure. BMC Public Health 23:415. doi: 10.1186/s12889-023-15309-3, PMID: 36859251 PMC9979418

[ref25] Tombul I. Sari G. (2021). Transformation of self presentation in virtual space. İletişim Kuram Ve Araştırma Dergisi 2021, 93–108. doi: 10.47998/ikad.852841

[ref26] Zimmermann D. Wehler A. Kaspar K. (2023). Self-representation through avatars in digital environments. Curr. Psychol. 42, 21775–21789. doi: 10.1007/s12144-022-03232-6

