# Supplementary material for: Extending the game immersion questionnaire to online users
Source: Front Psychol. 2025 Feb 11;16:1473821. doi: 10.3389/fpsyg.2025.1473821 (PMC11850392; doi:10.3389/fpsyg.2025.1473821)
Supplement: Supplementary file 1 [file Data_Sheet_1.docx]

Appendix 1: The Immersion in the Digital Environment Questionnaire to Online Users

WHEN CYBER ME IS ME!

We are inviting you to participate in innovative and interesting research about the digital identity of an individual. The research is conducted within the framework of a doctoral thesis, by Valērijs Dombrovskis ([valerijs.dombrovskis@kiberprats.lv](mailto:valerijs.dombrovskis@kiberprats.lv)) – a representative of Cyberpsychology Association, in cooperation with Daugavpils University, under the supervision of the professor Aleksejs Ruža. The survey consists of three parts:

1. introductory information about the research; 2) demographic data – general information about users of technologies; 3) test – questions about you and a particular digital space (a particular social media, online game, video hosting etc.) where your Cyber me lives.

**What is the purpose of this research and who conducts it?**

The given research is aimed at the investigation of the perception of **Cyber Me** by people. The term ‘Cyber Me’ relates to the part of our identity, which is reflected in cyber environment (digital profile, an online character/ video game character, avatar, etc.). It is interesting for us to find out when **Cyber Me** becomes a part of its creator.

**Participants**

In order to participate in this research, you have to be: 1) a user of technologies and 2) you have to have your **Cyber Me** (digital profile, video game character, etc.), which you actively use to communicate/ interact with other users. For instance, you publish some content in social media or play online games. An obligatory condition is as follows: your Cyber Me has to contain a visual component (an image, embodiment, picture, avatar, etc.), which you perceive when communicating/ interacting with other users.

**Participation and its Termination**

Participation in this research is absolutely voluntary and you can interrupt your being in it at any time. In case you want to finalise the participation, simply stop answering questions. However, we kindly ask you to complete all parts of the survey, not missing a single question. Your participation is very important. If you still refuse to take part in it, those materials you will have already filled in, will be deleted and will not be included in the research.

**Privacy and data Safety**

Information about you is confidential and your data will be coded by an identification code. Processing and storage of the information provided by you will be performed in compliance with the ‘Law of personal data protection’. Your provided data will be used only for research purposes.

**Ethical Issues and Contacts**

The present study was approved in accordance with the processes of ethical control of Daugavpils University. In case you would like to discuss the research or questions related to your participation in it, please contact one of research members: Valērijs Dombrovskis (valerijs.dombrovskis@kiberprats.lv; +37127003584) or Aleksejs Ruža (aleksejs.ruza@du.lv).

If you would like to find out about the results of the research you are participating in, you are free to contact us via e-mails indicated above after 1st January, 2023 and we will be pleased to send you a summary of the study and the obtained results.

Please read the instructions as well as questions carefully. There are no right or wrong answers – we are interested in your opinion. If you possess several profiles, which you use to communicate within digital environment, then choose one of them, the one reflecting your true identity, even it does not resemble you outside the digital environment.

First, we would like to ask you some questions about you:

**General information about a participant:**

1. **Gender:**

- M
- F
- Nonbinary

1. **Age (age last birthday): _____**
2. **My Cyber Me was created in:**

- Social media (for instance, Instagram, Facebook, Twitter)
- Online game
- Video hosting (for instance, YouTube, Tik Tok)

*Questionnaire Items*

| Item |  |
| --- | --- |
|  | **Engagement** |
| A1 | I would like to spend more time in digital space |
| A2 | I like the appearance and style of the digital space |
| A3 | I like being in the digital space because it is new and interesting |
| A4 | Generally, I can handle all the difficulties associated with being in the digital space |
| A5 | It is easy for me to control all the processes of the digital space |
| A6 | The user interface of the digital space makes me feel comfortable |
| A7 | I like the type of the digital space |
| A8 | I would like to spend time collecting the information of the digital space and discussing it with friends |
| A9 | The time I spend being in the digital space is always more than I expected |
|  | **Engrossment** |
| B1 | My ability to perceive the environment surrounding me is decreased while I am in the digital space |
| B2 | I am impatient when someone interrupts me when I am in the digital space |
| B3 | When I am in the digital space, I often cannot hear people who call me |
| B4 | I often feel nervous or excited because of the digital space |
| B5 | I often forget the passage of time while I am in the digital space |
| B6 | It frequently happens that I forget my schedule and/or to-do things in the real world while I am in the digital space |
| B7 | While I am in the digital space, I feel unhappy if someone interrupts me |
|  | **Total Immersion** |
| C1 | While I am in the digital space, it seems to me that everything that happens there, happens to me |
| C2 | My consciousness completely transfers from the real world to the digital space while I am solving the problems or tasks in the digital space |
| C3 | I lose the perception of time and the real world surrounding me, as if everything just stops |
| C4 | I feel happy or sad depending on what happens to my Cyber Me and sometimes I even feel that it exists |
| C5 | I used to be so integrated into the Cyber Me in the digital space that I could feel his/her feelings |
| C6 | All of my senses, including vision, learning, and my mind, are concentrated on and engaged in the digital space |
| C7 | I lose the ability of perceiving the surroundings around me; however, it seems natural for me to be totally immersed in the atmosphere of the digital space |
| C8 | I used to feel that the Cyber Me in the digital space is controlled by my will, and not by the mouse or the keyboard, so that the Cyber Me does just what I want to do. It seems like the thoughts and consciousness of the Cyber Me and Me are connected |

Note 1. Underlined items were deleted after EFA.
